# No Surprises Act Independent Dispute Resolution: A Five-Gate Evidentiary Framework for Evaluating Reform Claims

**DOI:** 10.7759/cureus.116646

**Published:** 2026-09-21

**Authors:** Andrew M Klapper, Anthony N Dardano, Michael Risin, Taylor Florio, Martha Denavea

**Affiliations:** 1 Plastic and Reconstructive Surgery, Delray Medical Center, Delray Beach, USA; 2 Nursing, Revenue Cycle, and Clinical Documentation, Floridian Institute of Plastic Surgery, Delray Beach, USA

**Keywords:** cms, federal idr, health policy, independent dispute resolution, medical arbitration, no surprises act, out-of-network payment, provider payment, qualifying payment amount, surprise billing

## Abstract

Background: The No Surprises Act (NSA) removes patients from many out-of-network payment disputes. Debate continues over whether the Federal Independent Dispute Resolution (IDR) process is being abused and should be restricted or rebenchmarked. We evaluate whether commonly cited Federal IDR statistics support the policy inferences drawn from them.

Methods: We apply a five-gate evidentiary framework (examining the Denominator, Comparator, Selection, Causation, and Attribution) to 12 recurring Federal IDR reform claims using statutory, regulatory, judicial, federal, and published sources. We supplement this framework with a line-item analysis of Centers for Medicare & Medicaid Services (CMS) Federal IDR public-use files for the first half of 2025.

Results: Federal IDR utilization greatly exceeds the original forecast of 22,232 disputes annually. CMS reports 6,336,032 disputes initiated through May 2026, of which 1,066,645 (16.83%) are found ineligible. Challenge rates represent a distinct and materially higher quantity, running approximately 40%-42% across individual CMS reporting periods, each with its own denominator. CMS reports that providers prevail in 87% of non-default, fully contested determinations in the first half of 2025. Among 1,694,788 analyzed line items, providers prevail in 1,476,967 (87.15%), and 1,446,720 (97.95%) of provider-prevailing line items have payments above the Qualifying Payment Amount (QPA). Among these same line items, however, 97.67% of all provider offers exceed QPA regardless of outcome, indicating that the observed coupling between provider victory and above-QPA payment is substantially consistent with the underlying positioning of provider offers. This observation does not explain the underlying win-rate asymmetry. The primary specification showed little evidence of a material residual association after stratification (Mantel-Haenszel odds ratio (OR) 1.0064, 95% cluster-bootstrap confidence interval (CI) 0.973-1.045; 2,000 replicates), as did a wider sensitivity specification (OR 0.9948, 95% CI 0.961-1.032). An exact-QPA post-hoc sensitivity specification showed a small positive association (OR 1.0367, 95% CI 1.002-1.080). Because multiple related sensitivity specifications were examined without multiplicity adjustment, this marginal estimate is exploratory rather than confirmatory. The cause of the overall adjudicatory asymmetry remains unresolved.

Conclusions: The examined statistics establish substantial Federal IDR utilization, ineligibility, filing concentration, adjudicatory asymmetry, and payment divergence from QPA, but do not independently establish provider abuse, benchmark validity, network erosion, price or premium inflation, or incremental arbitration cost. Applied symmetrically, the same evidentiary standard that prevents provider win rates from proving provider virtue also prevents those rates from proving provider abuse. Policy decisions increasing QPA’s determinative role require evidence capable of distinguishing case selection, payer behavior, benchmark performance, statutory factors, and adjudicatory effects.

## Introduction

In July 2026, policy analysis moved from the newest Federal Independent Dispute Resolution (IDR) data to a legislative prescription in a single chain: millions of disputes were treated as evidence of a system being exploited [1,2], provider victories and awards several times the Qualifying Payment Amount (QPA) were treated as evidence of arbitrators failing to anchor to the statutory benchmark [3,4], concentrated filing by a handful of organizations was treated as evidence of coordinated overuse [1,2], and billions in award and administrative costs were projected to reach patients through higher premiums and slower wage growth [5,6]. The proposed remedy was to replace individualized arbitration with QPA-based rate benchmarking. Each step is plausible, but none is the same empirical question as the previous one. A descriptive statistic does not acquire behavioral or causal meaning merely because several similarly directional statistics are presented beside it.

The No Surprises Act (NSA) solved a real problem: patients receiving emergency care, or care from nonparticipating clinicians at in-network facilities, should not be financial intermediaries in disputes between insurers and physicians [7]. Congress removed patients from many of these disputes and capped their cost-sharing exposure [7]. That is a settled success and not the subject of this paper. What Congress did not settle is how two sophisticated parties (an insurer and a nonparticipating provider) resolve a price disagreement once the patient is out of the picture. The statute created the Federal IDR process: a final-offer arbitration in which a certified independent entity considers the QPA and other statutory factors and selects one party’s offer [7]. It is not required to select the QPA, and it is barred from considering Medicare, billed charges, or usual-and-customary charges [7].

The evidentiary asymmetry

A recurring pattern is visible across the statistics used to support Federal IDR reform: a provider win rate is treated as evidence of provider misconduct [1-4], while an equally relevant evidentiary question is not addressed with the same evidentiary weight, namely whether the same loss rate, from the payer’s side, warrants investigation of payer-offer calibration, QPA construction, or adjudicatory mechanisms. Awards above QPA are treated as evidence of arbitrator or provider excess [2-4], while the accuracy of the payer-calculated QPA itself is frequently treated as an established baseline rather than an independently validated one [4]. Concentrated filing by a subset of provider organizations is treated as evidence of gaming [1,2] without the exposure denominator required to reach that conclusion for either provider or payer conduct. This paper’s normative position is that claims adverse to physicians should not receive a lower evidentiary threshold than competing explanations involving payer offer behavior, payer-constructed benchmarks, selection effects, or adjudicatory mechanisms. Instead, there should be a symmetric evidentiary burden. This is a claim about the standard that should govern inference, not a claim about which side is empirically correct, and the paper’s own strongest hypothesis favorable to physicians is tested against it directly, as described in the Results section.

Three claims should be kept distinct throughout what follows. The normative claim is that both sides of this debate should face the same evidentiary burden. The empirical claim is that commonly cited Federal IDR statistics frequently do not satisfy the inferential requirements necessary for the stronger conclusions attached to them. This paper tests that claim directly and reports even where the result is unfavorable to physicians. The open empirical question is which mechanisms actually explain the observed adjudicatory outcomes. This paper does not resolve that question, treating it as the field’s next task rather than as something directly answered by the presented framework.

We use a general-purpose analytic framework, which we call the Five-Gate Evidentiary Framework, to test the empirical claim. We developed this framework for the present analysis; it is not adapted from an existing published instrument. The framework organizes established principles of evidence appraisal, including denominator specification, comparator validity, selection, causal inference, and attribution, into a common structure for evaluating transitions from descriptive statistics to stronger policy conclusions. Its contribution is organizational rather than the creation of new principles of causal inference. The framework is proposed rather than validated, and its reliability, completeness, and usefulness beyond Federal IDR require independent evaluation. The five gates are as follows: Denominator, Comparator, Selection, Causation, and Attribution. The subsequent text defines each gate individually.

Study objectives

This study had three objectives. First, we developed and applied the Five-Gate Evidentiary Framework to organize the evidentiary requirements underlying major Federal IDR policy claims. Second, we evaluated whether publicly available Federal IDR statistics provide sufficient evidence to support 12 recurring claims concerning provider behavior, QPA validity, network participation, prices, premiums, and arbitration costs. Third, using CMS H1 2025 Federal IDR public-use data, we conducted an exploratory secondary-data analysis examining the relationship between payer-offer proximity to QPA and provider victory. The empirical component is associational and was not designed to establish causal effects of Federal IDR.

## Materials and methods

We conducted an original secondary-data analysis and structured evidentiary evaluation; this is not a causal econometric study. Primary sources included the NSA statutory text [7]; 2021-2026 federal rulemaking and regulatory impact analyses, including the 2026 Federal IDR Operations Final Rule [8]; and Centers for Medicare & Medicaid Services (CMS) Federal IDR public-use files and bi-monthly operational reports through May 31, 2026 [9]. Data periods differed according to the analysis performed. Descriptive Federal IDR utilization and operational statistics were evaluated through May 31, 2026, whereas the original line-item empirical analysis was restricted to CMS Q1-Q2 2025 public-use data. Judicial and regulatory developments through August 2026 were considered for legal and policy context. Findings from the H1 2025 line-item analysis should therefore not be interpreted as estimates for the full descriptive reporting period. Additional sources included Congressional Budget Office (CBO) [5] and Congressional Research Service (CRS) [10-12] analyses; federal judicial decisions on IDR implementation, including Texas Medical Association v. United States Department of Health and Human Services (HHS) (E.D. Tex., Feb. 23, 2022) [13] and subsequent related rulings [14]; QPA methodology and audit materials [15]; and peer-reviewed literature on Federal IDR outcomes [3,16]. Because [16] is an Elevance-affiliated analysis, it was treated as a payer-affiliated source rather than as independent confirmation of the related Elevance organizational analysis discussed later. Payer and industry advocacy statements were treated as claims under evaluation, not as evidence establishing those claims, and provider-favorable hypotheses were held to the same standard.

**Figure 1:**
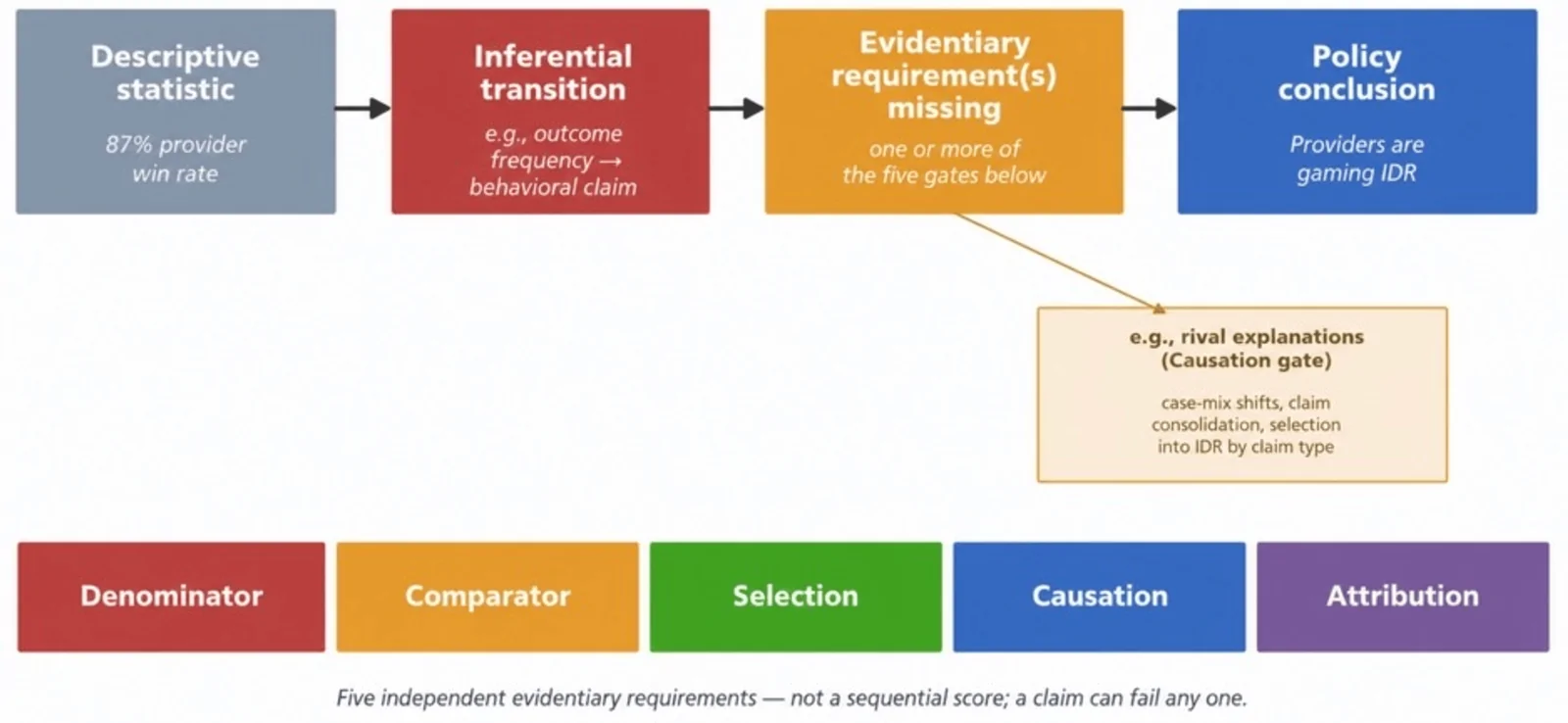
Example application of the Five-Gate Evidentiary Framework Schematic of the inferential path by which a descriptive statistic (e.g., an 87% provider win rate) is converted into a policy conclusion (e.g., “providers are gaming IDR”), with the five evidentiary gates- Denominator, Comparator, Selection, Causation, Attribution- shown as the set of requirements a given inferential transition may implicate. The gates are not sequential hurdles that every claim must clear in order; different claims implicate different gates, and a claim can be unsupported by failing to satisfy even one relevant gate. The inset at the Causation gate lists named rival explanations (case-mix shifts, claim consolidation, selection into IDR by claim type) as an illustration of the alternative-explanation requirement that gate imposes; it is not an exhaustive list. The figure does not classify any specific claim in this debate as true or false; it identifies the evidence a given inference requires. The Five-Gate Evidentiary Framework was developed by the authors for the present study and is not an externally validated instrument. IDR: Independent Dispute Resolution

We examined 12 recurring claims using the five inferential questions above. The Five-Gate Framework is a proposed structured analytic framework, not a validated scoring, classification, or measurement instrument (Figure 1). It assembles denominator specification, comparator validity, selection, causal identification, and attribution into a common structure for evaluating payment-policy claims. These components reflect established principles of epidemiology, observational inference, and evidence appraisal rather than newly proposed principles of causal inference. The framework has not been externally validated, has not been shown to be exhaustive or superior to alternative evidence-appraisal approaches, and has not been tested for inter-rater reliability. We did not assign numerical gate scores or interpret the number of implicated gates quantitatively. In addition, CMS does not currently publish a national denominator of federally IDR-eligible out-of-network claims. This absence limits every claim evaluated in this paper as much as it limits the claims under evaluation.

Supplemental line-level empirical analysis

To test whether provider-prevailing determinations and above-QPA payments represent substantially overlapping rather than merely parallel populations, we conducted a line-item analysis of CMS’s Q1-Q2 2025 Federal IDR public-use files [9], comprising 1,694,788 non-default line items with usable offer/QPA fields from 792,790 underlying disputes. The line item was the principal analytical unit because provider offer, payer offer, QPA, selected payment, service code, and prevailing party are reported at the line-item level and may differ among multiple line items within the same dispute. Collapsing such disputes to a single observation would therefore require aggregation of substantively distinct payment comparisons. Provider victory was defined as the selection of the provider/facility/air-ambulance-provider offer. Above-QPA payment was defined as selected payment/QPA > 1.00. Payer-offer proximity was defined as payer offer/QPA. The primary specification classified ratios from 0.95 through 1.05 as in-band; a wider post-hoc sensitivity specification classified ratios from 0.90 through 1.10 as in-band; and an exact-match post-hoc sensitivity specification required payer offer/QPA = 1.00. We estimated Mantel-Haenszel odds ratios using strata defined by the cross-classification of quarter × exact CMS-reported payer name × service code × provider-offer/QPA band. Exact CMS-reported payer strings were retained after Extensible Markup Language (XML) unescaping; no payer-name canonicalization was performed, so naming variants remained separate strata. No payer-name-normalized analysis was performed. An informative stratum was one containing observations capable of informing estimation of the common odds ratio under the specified comparison. Because multiple line items within the same dispute are not statistically independent, 95% confidence intervals were estimated using cluster-bootstrap resampling of underlying disputes with replacement rather than resampling individual line items. We used 2,000 bootstrap replicates. All statistical analyses were performed in Python 3.13.5 (Python Software Foundation, Wilmington, Delaware, USA), using SciPy 1.17.0 (SciPy Developers; NumFOCUS, Austin, Texas, USA) and statsmodels 0.14.6 (statsmodels Developers; NumFOCUS, Austin, Texas, USA). The analytic cohort and deterministic stratification pipeline were subsequently reconstructed from the original CMS source workbooks using the preserved extraction and stratification code. The reconstructed analytic Comma-Separated Value (CSV) files and principal stratified-result artifact matched the preserved originals exactly by Secure Hash Algorithm 256-bit (SHA-256) comparison. The original bootstrap implementation and random seed were not recoverable; an independent implementation of the described dispute-level cluster-bootstrap procedure produced confidence intervals closely concordant with the reported values across multiple random seeds. No timestamped protocol establishes prespecification of the wider-band or exact-match analyses; both are therefore identified throughout as post-hoc sensitivity analyses. Because multiple related specifications were examined without adjustment for multiplicity, any marginal association observed in these sensitivity analyses is considered exploratory rather than confirmatory.

Table 1 summarizes the 12 recurring Federal IDR policy claims examined in this study, the descriptive evidence associated with each, the inferential step being made, and the additional evidence required to support that inference.

**Table 1:** Evidentiary requirements for common Federal IDR policy inferences Summary of the 12 recurring Federal IDR policy claims evaluated using the Five-Gate Evidentiary Framework. For each claim, the table identifies the descriptive evidence available, the inferential step required to support the stronger policy conclusion, the additional evidence needed, and what can currently be concluded. Failure to satisfy an evidentiary requirement does not establish the opposite conclusion. Sources synthesized: CMS data [9], CBO [5], CRS [10-12], TMA rulings [13,14], QPA audit findings [15,17], and peer-reviewed literature [3,16]. CMS: Centers for Medicare & Medicaid Services; CBO: Congressional Budget Office; CRS: Congressional Research Service; TMA: Texas Medical Association; QPA: Qualifying Payment Amount; IDR: Independent Dispute Resolution

Claim No.	Policy claim	Descriptive evidence available	Inferential step being made	Additional evidence required	What can currently be concluded
1	Millions of disputes prove abuse	CMS reports 6,336,032 disputes initiated through May 2026	Volume → abuse rate	Eligible-claims denominator (not publicly available)	Utilization established; abuse rate cannot currently be estimated
2	“Upwards of 40%” ineligibility	CMS: ~40–42% challenged; ~17–19% found ineligible in individual reporting periods (period-specific denominators)	Challenge rate treated as ineligibility rate	Correct denominator (determinations, not challenges); reason-specific breakdown	1,066,645 of 6,336,032 disputes initiated through May 2026 (16.83%) have been found ineligible; provider misconduct not established either way
3	Filing concentration proves gaming	Top organizations file large dispute shares	Share of filings → excess filing intensity	Exposure-adjusted denominator (eligible claims per organization; not publicly available)	Concentration established; excess intensity cannot currently be estimated in either direction
4	Provider win rate proves misconduct	87% non-default win rate (H1 2025)	Outcome frequency → valuation/behavioral judgment	Data distinguishing case-selection, benchmark-inadequacy, and adjudicator-effect explanations	Asymmetry established; its cause is not established
5	Provider win/above-QPA coupling proves benchmark failure	1,446,720 (97.95%) of provider wins have award > QPA	Coupling → independent significance beyond offer positioning	Offer-positioning baseline (tested); stratified test of the QPA-proximity mechanism (tested)	Provider wins, and above-QPA payments substantially overlap at the line-item level; this does not establish that QPA divergence proves benchmark invalidity or that payer proximity to QPA materially explains provider victory—primary and wider-band specifications showed little evidence of a material association, while the exact-match sensitivity analysis showed a small association
6	Medicare multiples prove excess payment	Awards reported as multiples of Medicare	Distance from Medicare → commercial economic excess	A validated commercial-value comparator (Medicare is excluded by statute from adjudication)	Descriptive distance established; economic excess not established (nor is a large multiple thereby shown to be reasonable)
7	IDR causes network exit	CBO: participation increased, preliminary	Current trend → causal claim about IDR	Market-level causal design	Direction (participation up) established at preliminary strength only; causal claim not established
8	IDR causes negotiated-price inflation	CBO: prices generally declining, preliminary	Theoretical mechanism → observed effect	Market-level IDR-intensity/price causal design	Hypothesis only
9	IDR causes premium inflation	CBO: evidence mixed; a contemporary example (Elevance) applies this claim to a selected cohort with no described premium-incidence model	Long causal chain asserted as established	Multi-year premium/IDR-intensity panel design; counterfactual-based incidence modeling	Hypothesis only; award-to-benchmark divergence is not itself a demonstrated premium effect
10	Aggregate awards equal new healthcare cost	Gross award totals reported	Gross payment → incremental cost	A valid non-arbitration counterfactual payment estimate	Gross totals established; incremental cost not established
11	QPA departure proves arbitrator malfunction	Awards frequently exceed QPA	Departure from QPA → departure from statutory criteria	Evidence the adjudicator disregarded, rather than weighed, the statutory factors	Not established; QPA is one statutory factor among several, not a required outcome
12	Provider conduct constitutes gaming	Providers select cases and submit offers	Filing behavior → control over outcome	Evidence distinguishing provider-controlled conduct from certified-IDR-entity selection	Not established as stated; requires IDR-entity-level analysis

## Results

Denominator problems

Volume

**Figure 2:**
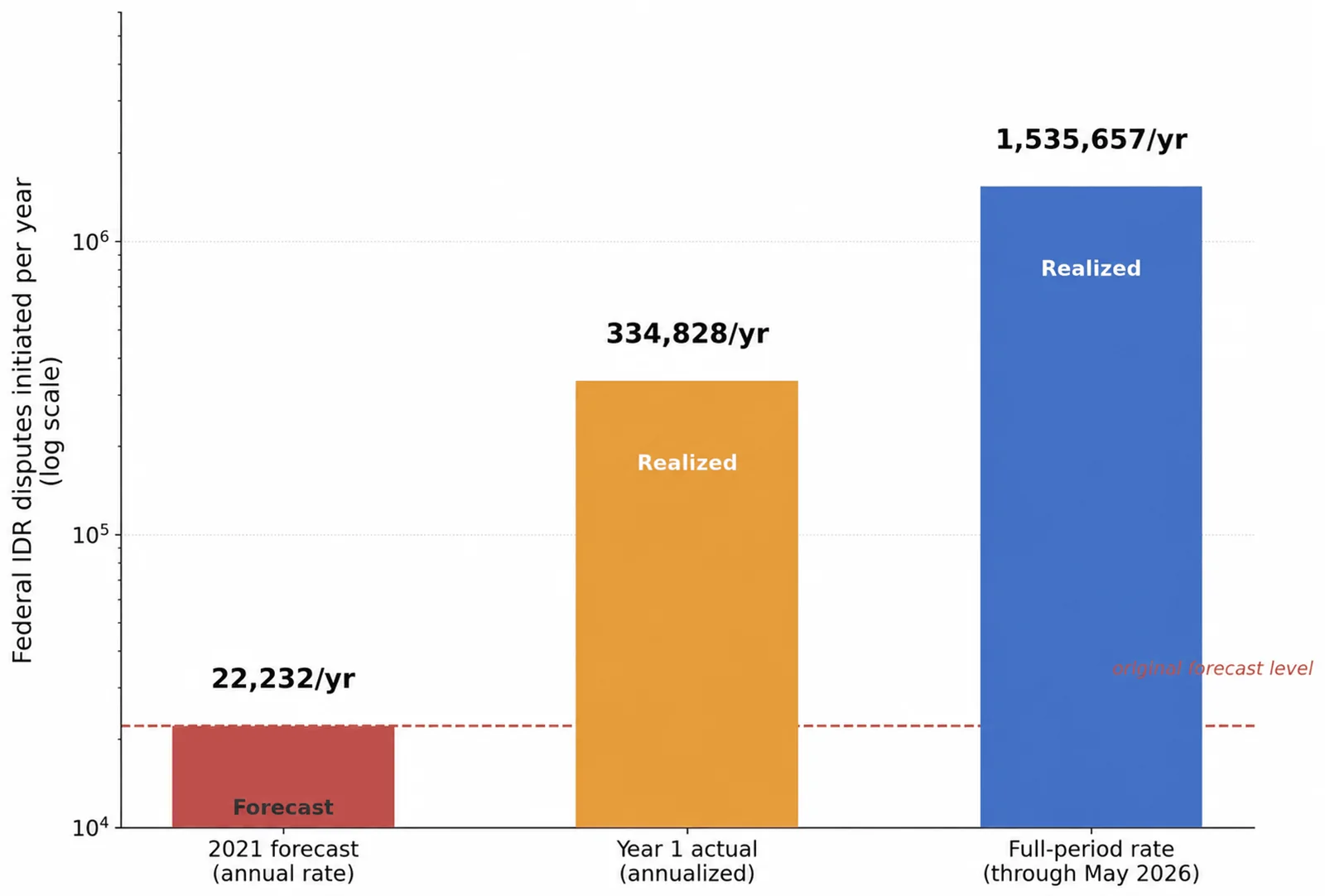
Federal IDR utilization compared with the original administrative forecast Comparison, on an apples-to-apples annualized basis, of the original 2021 administrative forecast (22,232 disputes/year: 17,333 non-air-ambulance plus 4,899 air-ambulance) against Year-1 actual volume and the full- period annualized rate through May 2026 (6,336,032 cumulative disputes initiated, divided by elapsed program years); the intermediate 2023 revised paperwork estimate (420,000/year) is discussed in text. Annualized rates, not a cumulative total, are plotted against the original annual forecast to avoid an annual-versus-cumulative comparison error. The figure shows the magnitude of the difference between the original forecast and observed volume. Exceeding an administrative forecast by this margin does not, by itself, identify the cause of the excess volume. Data sources: original 2021 forecast [18]; 2023 revised estimate [19]; cumulative disputes through May 2026 [9]. IDR: Independent Dispute Resolution

The forecast of roughly 22,000 disputes per year (17,333 non-air-ambulance plus 4,899 air-ambulance, totaling 22,232) emerged from 2021 rulemaking under the acknowledged absence of federal data on the part of the Departments of Health and Human Services, Labor, and the Treasury (collectively, "the Departments," the agencies that jointly administer Federal IDR rulemaking [18]), extrapolated from roughly 1,000 New York decisions and New York’s estimated 5.8% share of the private insurance market [18]. It also assumed QPA would function as a rebuttable presumption suppressing volume by making outcomes predictable, an assumption that did not survive Texas Medical Association v. HHS (TMA I, Feb. 2022), which vacated the regulatory QPA presumption [13]. By 2023, the Departments’ own paperwork estimate had grown to 420,000 disputes annually [19]. CMS now reports 6,336,032 disputes initiated through May 2026 (Figure 2) [9].

Ineligibility

As of May 2026, 1,066,645 disputes had been found ineligible, equal to 16.83% of the 6,336,032 disputes initiated [9]. Individual 2025 reporting periods separately show ineligibility findings of approximately 17-19% [20,21]. These are period-specific figures and should not be assumed to share the same denominator as the cumulative 16.83% figure, though both describe disputes found ineligible rather than disputes merely challenged on eligibility grounds. Eligibility challenge rates in the same periods run approximately 40-42% [20,21], a materially different quantity, since a challenge is not a determination. United Healthcare’s Dan Kueter publicly stated on the company’s Q2 2026 earnings call that upwards of 40% of claims entering IDR are ineligible [6]. However, that figure does not disclose its underlying population or denominator and cannot presently be reconciled with the national data above. CMS's 2026 Federal IDR Operations Final Rule requires standardized payer claim-adjustment codes; CMS states these requirements address information-architecture failures (disputes filed before the statutory 90-day cooling-off period, or where a state law or All-Payer Model displaced federal jurisdiction), not solely provider conduct, and are intended to reduce ineligible submissions [8].

Filing Concentration

A small number of organizations account for a large share of 2025 filings. An eligible-claims-per-organization denominator, needed to assess whether that filing concentration is disproportionate to eligible exposure, is not publicly available.

Comparator problems

The denominator problems above concern how much is happening. The comparator problems below concern what a given outcome should be measured against, and whether that reference point has itself been validated for the conclusion being drawn from it.

Win Rate

CMS reports that providers, facilities, and air-ambulance providers prevailed in 88% of all determinations in the first half of 2025 and in approximately 87% of non-default, fully contested determinations [20]. For purposes of this manuscript, a default determination is one not resolved through a fully contested selection between competing offers; non-default, fully contested determinations are the subset in which the adjudicatory outcome reflects the contested offer-selection process. Our original line-item empirical cohort is restricted to non-default H1 2025 records and yields a provider win rate of 87.15% (1,476,967 of 1,694,788 line items). Accordingly, 87.15% is the primary empirical win-rate figure carried through our original analysis, while the CMS-reported 88% figure is retained only when describing CMS’s broader determination-level statistic. CMS reports an 85% provider win rate in the second half of 2025 [21].

QPA Divergence

Providers prevailed in approximately 87% of non-default determinations in the first half of 2025, and prevailing offers exceeded QPA in approximately 87-88% of cases [20,21]. Federal law requires certified IDR entities to consider the QPA, but responsibility for verifying whether it was calculated correctly rests with regulators, not with the provider or the certified IDR entity that must weigh it [7,8]. QPA calculation error is not merely theoretical. CMS reports initiating 25 QPA audits since June 10, 2022, of which one, involving Aetna Health-Texas air-ambulance claims, was concluded during calendar year 2024. It found five incorrectly calculated QPAs, with errors running in both directions [15,17]. A follow-up Aetna self-audit of 89 additional claims found no further errors [15]. QPA methodology also remains operationally unsettled. On August 11, 2026, the Fifth Circuit issued its en banc decision in Texas Medical Association v. United States Department of Health and Human Services, holding that the challenged QPA methodology was unlawful insofar as it permitted the inclusion of non-negotiated “ghost rates” and the exclusion of bonus and incentive payments, while rejecting the challenge concerning exclusion of certain one-off agreements. The court affirmed in part, reversed in part, and remanded [14]. The Departments have separately extended enforcement discretion for the 2021 QPA methodology through items and services furnished before October 1, 2026 [22].

Medicare Multiples

Congress explicitly barred IDR entities from considering Medicare, billed charges, or usual-and-customary charges [7]. A Brookings Institution analysis of IDR data found that emergency care lands close to its pre-NSA baseline, imaging runs roughly 2 to 2.4 times that baseline, and neonatal/pediatric critical care roughly 1.7 times, using pre-NSA out-of-network allowable amounts adjusted for inflation as the comparator [1]. The Niskanen Center has since cited this same Brookings comparison in arguing for legislative restrictions on Federal IDR [2].

Supplemental empirical analysis

The comparator problems above are argued from published aggregate statistics. The analysis below tests one of them directly at the line-item level: a hypothesis whose failure would support claims adverse to physicians and whose success would have favored physicians.

**Figure 3:**
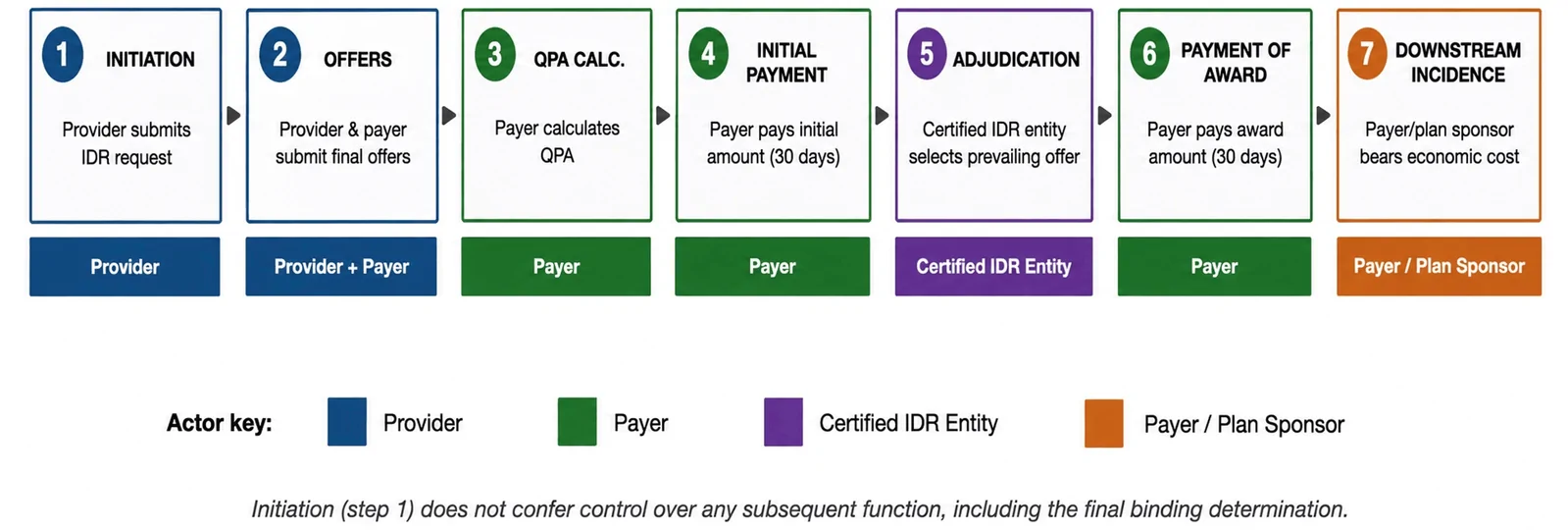
Functional attribution across the Federal IDR process Seven actor-controlled functions are shown from initiation through downstream incidence, with the responsible actor identified for each step. Initiation does not confer control over benchmark calculation, initial payment, adjudication, award payment, or downstream economic incidence. The authors’ schematic is based on CMS, CBO, CRS, and statutory/regulatory process descriptions [5,7,8,10-12]. CMS: Centers for Medicare & Medicaid Services; CBO: Congressional Budget Office; CRS: Congressional Research Service; IDR: Independent Dispute Resolution; QPA: Qualifying Payment Amount

A line-item analysis of 1,694,788 H1 2025 non-default determinations tested whether provider victories and above-QPA payments represent the same determinations rather than parallel statistics. Providers prevailed in 1,476,967 (87.15%) of line items, and selected payments exceeded QPA in 1,562,385 (92.19%). The two were strongly coupled: 1,446,720 (97.95%) of provider-prevailing line items had payments above QPA, and 1,446,720 (92.60%) of above-QPA payments were provider-prevailing (Table 2). This pattern was consistent across both quarters (Table 3). Figure 3 provides an overview of functional attribution across the Federal IDR process.

**Table 2:** Relationship between prevailing party and payment relative to the QPA, H1 2025 Provider win rate 87.15%; award > QPA rate 92.19%; P(award > QPA | provider win) 97.95%; P(provider win | award > QPA) 92.60%; P(award > QPA | payer win) 53.10%; P(provider win | award ≤ QPA) 22.84%. These are descriptive line-item proportions, not inferential statistics; no p-values are reported for this table. The 1,694,788 line items arise from 792,790 underlying disputes (mean 2.14, median 1 line item per dispute): 572,754 disputes (72.25%) are singletons contributing exactly one line item each (33.80% of all line items), while 220,036 disputes (27.75%) are batched, contributing 1,122,034 line items (66.20% of the total) that require clustering. Where this manuscript reports inferential uncertainty, it is computed via a cluster bootstrap resampling disputes, not line items. Present analysis of CMS Federal IDR public-use data [9]. QPA: Qualifying Payment Amount

Prevailing party	Award > QPA	Award ≤ QPA	Total
Provider/facility/air-ambulance provider prevails	1446720	30247	1476967
Plan/issuer prevails	115665	102156	217821
Total	1562385	132403	1694788

**Table 3:** Provider win rates and above-QPA payments by quarter, Q1–Q2 2025 Consistent direction and magnitude across both quarters; the primary result is not a single-quarter artifact. Present analysis of CMS Federal IDR public-use data [9]. QPA: Qualifying Payment Amount; CMS: Centers for Medicare & Medicaid Services; IDR: Independent Dispute Resolution

Quarter	N	Provider win rate	Award > QPA rate	P(> QPA | provider win)	P(provider win | > QPA)
2025 Q1	701940	86.86%	92.51%	98.37%	92.37%
2025 Q2	992848	87.35%	91.96%	97.66%	92.76%

We tested that reading directly. Among all line items, irrespective of outcome, 97.67% of provider offers exceeded QPA (median 4.37× QPA, IQR (2.72, 8.67)), against only 46.64% of payer offers (median 1.00×, IQR (1.00, 1.36)); 44.51% equaled QPA exactly. We have not formally decomposed the exact fraction attributable to offer positioning and do not claim to have done so.

**Figure 4:**
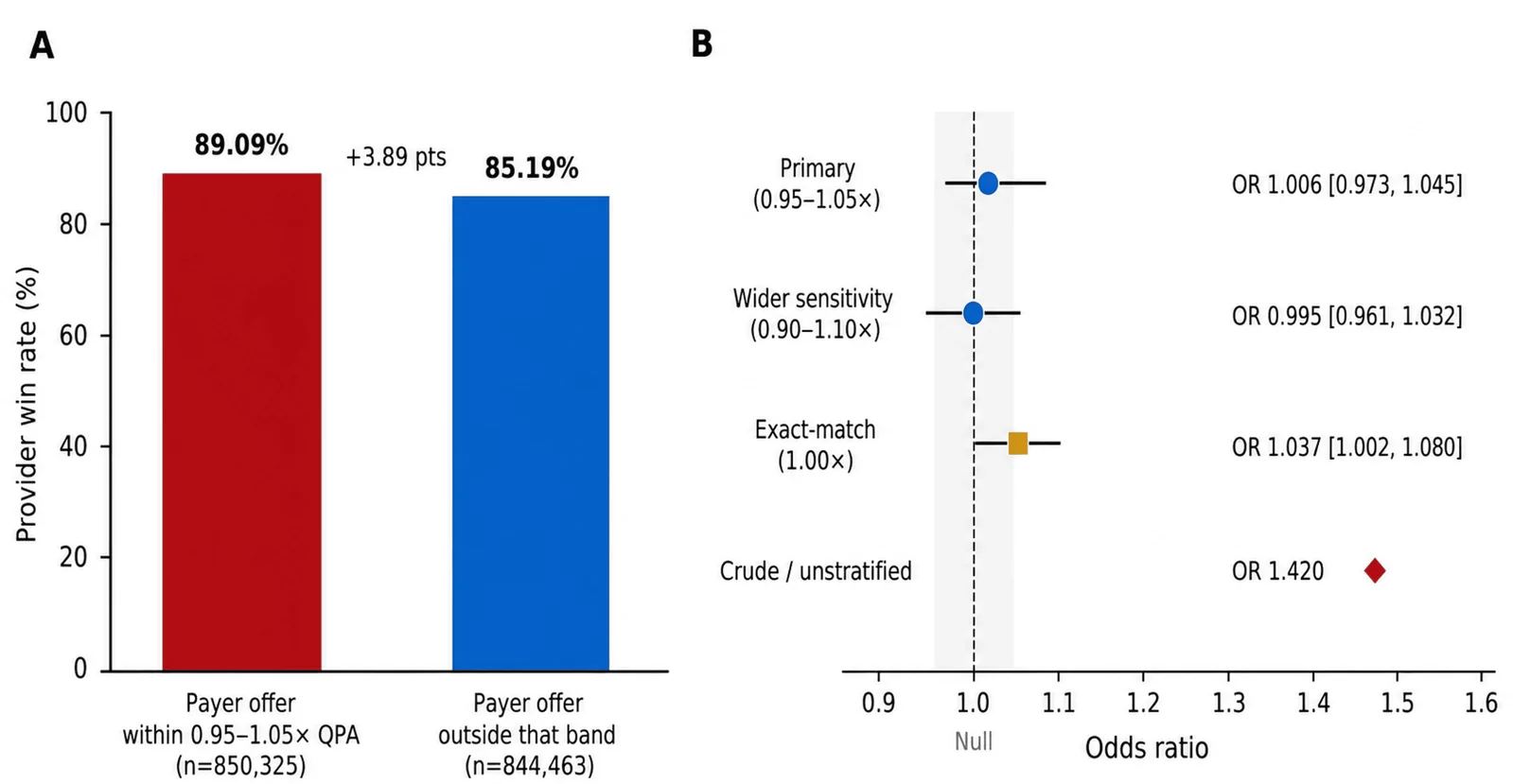
Crude and stratified associations between payer QPA proximity and provider victory Panel A shows the crude provider win-rate difference between payer offers within 0.95–1.05× QPA and offers outside that band (89.09% vs. 85.19%; +3.89 percentage points). Panel B shows the corresponding crude unstratified odds ratio (OR 1.420) alongside stratified Mantel-Haenszel estimates for the primary 0.95–1.05× specification (OR 1.006, 95% CI 0.973–1.045), the wider 0.90–1.10× sensitivity specification (OR 0.995, 95% CI 0.961–1.032), and the exact 1.00× sensitivity specification (OR 1.037, 95% CI 1.002–1.080). The dashed vertical line marks the null value (OR = 1.0). Confidence intervals were estimated by cluster bootstrap resampling underlying disputes. Present analysis of CMS Federal IDR public-use data [9]. CMS: Centers for Medicare & Medicaid Services; QPA: Qualifying Payment Amount; IDR: Independent Dispute Resolution

Providers prevail in 87.15% of the analyzed non-default line items despite provider offers sitting substantially farther from QPA (median 4.37×) than payer offers (median 1.00×). We tested the hypothesis that payer-offer proximity to QPA predicts provider victory. After stratification by quarter, exact payer name, service code, and provider-offer/QPA band (28,237 informative strata), the primary specification showed little evidence of a material residual association (MH OR 1.0064, 95% cluster-bootstrap CI (0.973, 1.045), 2,000 replicates) (Table 4). A wider post-hoc sensitivity specification likewise showed little evidence of a material association (0.90-1.10×; OR 0.9948, CI (0.961, 1.032)) (Figure 4). The post-hoc exact-match specification (payer offer precisely 1.00× QPA) showed a small positive association (OR 1.0367, CI (1.002, 1.080)). Because the latter two specifications were selected after the primary result and multiple related specifications were examined without adjustment for multiplicity, the exact-match finding is exploratory and should not be interpreted as a standalone confirmatory result. This is a stratified association analysis, not a causal identification strategy. It does not establish that QPA positioning has no causal effect on adjudication under a different design, nor does it rule out mechanisms operating through unobserved covariates such as acuity, contracting history, or evidence submitted to the arbitrator.

**Table 4:** Crude vs. stratified QPA-proximity result All specifications use exact CMS-reported payer strings without payer-name canonicalization; naming variants therefore remain separate strata. Confidence intervals were estimated via cluster bootstrap resampling of the 792,790 underlying disputes with replacement. The primary and wider-band specifications have confidence intervals spanning the null (1.0). The exact-match specification shows a small positive association but was a post-hoc sensitivity analysis, one of multiple related specifications examined without adjustment for multiplicity, and should therefore be considered exploratory rather than confirmatory. No payer-name-normalized analysis was performed. The present analysis used CMS Federal IDR public-use data [9]. CMS: Centers for Medicare & Medicaid Services; QPA: Qualifying Payment Amount; IDR: Independent Dispute Resolution

QPA band definition	N in-band win rate	N out-of-band win rate	Crude difference	MH OR	Informative strata	95% cluster-bootstrap CI (2,000 replicates)
0.95–1.05x (primary specification)	89.09% (n=850,325)	85.19% (n=844,463)	+3.89 pts	1.0064	28237	(0.973, 1.045)
0.90–1.10x (wider sensitivity specification)	88.85% (n=892,217)	85.26% (n=802,571)	+3.59 pts	0.9948	28800	(0.961, 1.032)
Exact 1.00x (exact-match sensitivity specification)	89.40% (n=754,389)	85.34% (n=940,399)	+4.05 pts	1.0367	26275	(1.002, 1.080)

Two limitations accompany this analysis. These are line-item, not dispute-level, estimates; within-dispute dependence is addressed through dispute-level cluster bootstrap rather than by treating each line item as independent. The finding is confined to the selected, adjudicated Federal IDR population and does not establish anything about QPA’s adequacy for the much larger population of commercial claims that never reach arbitration. Exact CMS-reported payer-name strings were used as published, apart from XML unescaping, and payer entities were not canonicalized across naming variants; economically related payer names may therefore remain divided among separate strata. No payer-name-normalized analysis was performed. The deterministic extraction and stratification pipeline was subsequently reproduced exactly from the original CMS source workbooks and preserved code, but the original bootstrap implementation and random seed were not recoverable. The reported bootstrap intervals were therefore independently corroborated rather than reproduced bit-for-bit.

The cause of the 87.15% win-rate asymmetry remains unresolved. Candidate explanations, including provider case selection, relative offer competitiveness, QPA performance, payer strategy, statutory-factor evidence, case mix, and adjudicator effects, remain open, singly or in combination.

Causal and attribution problems

The sections above concern how much is happening and what it should be measured against. The two below concern a different question: even where an effect is real, has it been shown to be caused by Federal IDR, and by which actor’s conduct? Descriptive magnitude is not causal identification.

Network Participation, Prices, and Premiums

CBO reports preliminary evidence of increased network participation and a declining out-of-network claim share since the NSA's implementation, and that available studies generally show declining inflation-adjusted prices for several affected services. It also explicitly requests new market-level research on both questions [5]. Roughly 80% of the NSA’s originally projected savings were expected to come from lower in-network prices, driven by a weakened provider out-of-network bargaining alternative [5]. CBO characterizes the evidence on premiums as mixed [5]. The Niskanen Center’s July 2026 analysis states that, without reform, patients will “continue to bear these costs, not through the surprise bills the law was designed to eliminate, but through the higher premiums and slower wage growth that follow when insurers pass their higher costs to customers” [2], while CBO’s contemporaneous assessment treats the same question as unresolved and names the research design that would resolve it [5].

Aggregate Cost

The Niskanen analysis reports a $2.8 billion total-program-cost figure and a $1.2 billion IDR-entity-compensation figure for 2025 [2].

Attribution

At least seven distinct functions are involved in a single Federal IDR determination, and these are frequently performed by different actors (Figure 3). The provider initiates the dispute, the provider and payer each submit an offer, the payer calculates the QPA, the payer sets the initial payment, the certified IDR entity selects the prevailing offer, the provider receives the resulting payment, and some combination of payer, employer plan sponsor, and ultimately enrollees may bear the downstream economic incidence, a question this paper’s Causation results leave open. CMS’s 2026 Federal IDR Operations Final Rule redesigns payer-to-provider information exchange specifically to reduce ineligible disputes [8].

A 99.9% provider-initiation share is reported consistently across 2025 reporting periods [2]. CMS's newly available certified IDR entity identifier field was added administratively in the routine Q3/Q4 2025 public-use file release [21].

## Discussion

Interpreting the results

Three findings in the Denominator results carry interpretive weight beyond the numbers themselves. First, on utilization, the original 22,232-dispute forecast was an ex ante estimate built without national data, under a regulatory architecture the courts later vacated in TMA I [13]. It is not a normative baseline for legitimate use. When observed volume exceeds a forecast this badly, the first established conclusion is that the forecast failed, not that participants misbehaved. Second, on ineligibility, collapsing challenge rates into determination rates, or determination rates into a provider-misconduct rate, are two separate inferential errors. Both happen to support the same conclusion: that the ineligibility problem is larger and more behaviorally attributable to providers than the underlying data establish. CMS's own information-architecture rationale for its 2026 standardized-code requirement indicates that a meaningful share of ineligibility is procedural rather than behavioral, reinforcing this point [8]. Third, on filing concentration: this statistic is frequently treated as evidence of gaming [1,2], but the evidentiary question that treatment skips is whether filing is disproportionate to eligible exposure. A large multispecialty group generates more eligible disputes than a small practice on scale alone, and centralized dispute infrastructure lowers the marginal cost of pursuing each additional legitimate claim. Without an eligible-claims-per-organization denominator, this paper cannot establish that filing is disproportionate or proportionate; the missing denominator constrains claims of behavioral misconduct and this paper’s ability to rule such claims out in equal measure.

The Comparator results raise a parallel set of interpretive questions. A win rate is an outcome frequency, not a valuation judgment, yet the prevailing framing treats the 87-88% provider win rate as a question about provider conduct [1-4]. An equally relevant evidentiary question is whether the same loss rate, from the payer’s side, warrants investigation of payer-offer calibration, QPA construction, case selection, statutory factors, or adjudicatory mechanisms. Final-offer arbitration disciplines offers through the incentive to avoid a less-supportable position, but it does not guarantee self-correction if both offers are poorly calibrated or if adjudicators behave systematically in one direction. A win rate this lopsided is consistent with several mechanisms, including provider selection of strong cases, systematically low payer offers, benchmark inadequacy, statutory factors, adjudicator effects, or combinations of these; the aggregate statistic cannot distinguish among them. Provider self-selection for strong cases remains a viable partial or complete explanation, but its contribution cannot be quantified because claims eligible for Federal IDR but never initiated are not publicly observable. We do not claim payer misconduct from this asymmetry. Doing so would commit the same inferential error this paper identifies in claims that treat the win rate as evidence of provider misconduct. The point is that this asymmetry, for which only one side’s conduct is scrutinized, is unexplained by the data.

The QPA-divergence findings invite a similar reframing. The 87-88% rate at which prevailing offers exceed QPA is commonly read as evidence that providers or arbitrators are producing excessive awards [2-4,23]. The symmetric question is why the payer-calculated QPA’s own accuracy and economic relevance is so often treated as an established baseline rather than something requiring independent validation. The confirmed QPA-calculation errors reported above establish the existence of QPA calculation error in operational practice. They do not establish prevalence, systematic undercalculation, or payer misconduct, and this paper does not claim otherwise. The ghost-rate controversy discussed above, in which the Fifth Circuit held part of the QPA methodology unlawful [14], provides a concrete example of the Comparator gate: the existence of a calculable benchmark does not establish the economic validity of the transactions embedded within it.

The Medicare-multiple comparison is subject to the same symmetric-error principle. Reporting an award as a multiple of Medicare is a mathematical comparison; treating that multiple as evidence of commercial economic excess requires Medicare to be the correct commercial valuation denominator, which is precisely the assumption Congress declined to adopt for this process. The error runs in the other direction as well. A large Medicare multiple does not, by itself, establish that an award is reasonable. It shows only that Medicare, a price Congress excluded from adjudication, is not the applicable reference. The pre-NSA-baseline comparison reported above references a real historical price rather than an administered or statutorily excluded one, though it still does not by itself supply the Selection, Causation, or Attribution evidence that downstream remedy claims require. The Niskanen Center’s subsequent use of this same Brookings comparison to argue for legislative restrictions on Federal IDR illustrates how an empirical finding can be carried into policy advocacy substantially unchanged in its numbers but advanced toward a stronger conclusion than the underlying analysis itself draws.

The line-item coupling result also requires interpretive care. The strong overlap between provider victories and above-QPA payments could otherwise be interpreted as an independent adjudicatory phenomenon. However, 97.67% of provider offers themselves exceed QPA, while 97.95% of provider-prevailing line items have above-QPA payments. The near-identity of these proportions indicates that the observed coupling is substantially consistent with the underlying positioning of provider offers and should not, by itself, be interpreted as evidence of an independent adjudicatory mechanism producing above-QPA payments. This observation does not explain the underlying provider-payer win-rate asymmetry. Providers prevail in the large majority of contested determinations despite their offers sitting substantially farther from QPA than payer offers, and the cause of that outcome asymmetry remains unresolved.

Finally, the Attribution results warrant a caution about what a provider-initiation share can and cannot show. “Provider gaming” as a claim typically attributes the final adjudicated outcome to the party that initiated the dispute, even though that party does not select the winning offer [1,2]. Visibility is not attribution: the actor most visible in the transaction is not necessarily the actor who controlled the result. An allegation that providers are exploiting the process collapses several of the seven distinct functions identified above into one actor’s control. It requires evidence distinguishing provider-controlled conduct (initiation, offer submission) from certified-IDR-entity-controlled outcomes (selection), which is evidence the aggregate win rate alone cannot supply. CMS’s 2026 Federal IDR Operations Final Rule [8] is itself a government acknowledgment that some outcomes originate in information architecture rather than exclusively in provider conduct.

The Causal results on network participation, prices, and premiums also require care in interpretation. If Federal IDR preserves some of the provider out-of-network bargaining alternative the NSA was designed to weaken, that is not automatically evidence of malfunction. On premiums specifically, the causal chain runs from award to provider leverage, network and out-of-network spending, insurer cost, and finally premium. This is the longest chain in this debate, consistent with CBO's characterization of the evidence as mixed and preliminary [5].

The aggregate cost figures reported above also require disaggregation before they support a cost claim. Treating gross IDR award totals as new healthcare costs requires assuming the counterfactual payment for the same services, absent arbitration, would have been zero. At least five distinct quantities are routinely conflated. These are the gross award or payment flow, its increment over the insurer’s initial payment, its increment over QPA, the increment over the actual counterfactual payment absent Federal IDR (which alone approximates the payment attributable to the arbitration mechanism itself), and administrative or resource costs such as arbitrator fees and processing costs, which are real and separately measurable. Downstream premium incidence is a sixth, further question this paper does not resolve. Even the mechanism-attributable increment, once identified, is initially a transfer between payer and provider rather than a self-evident societal loss. The Niskanen figures reported above are administrative and adjudication expenditures in this narrower sense, not incremental medical-service costs.

Nor does the initiation share reported above cut only in one direction. It establishes who invokes the remedy, not why that party subsequently prevails, since a party that never initiates cannot win. Who invokes a remedy is not the same question as who controls its outcome. A per-determination certified-IDR-entity fee structure creates an incentive worth examining, but it does not show that the incentive altered any specific selected offer. We do not treat either fact as proof of payer or adjudicator responsibility. An observed payment outcome is jointly produced by provider selection and offer-setting, payer payment and benchmark construction, statutory decision criteria, and certified IDR adjudication. Attributing the resulting asymmetry to one actor requires evidence identifying which of those functions generated the observed result. The newly available certified IDR entity identifier field makes the adjudicator-effects question directly testable via case-mix-adjusted, entity-level analysis. This is the highest-value next step available for resolving it.

Contemporary applications of snippet-to-policy conversion

The 12 claims above are not abstractions. They are being made, in these terms, in current policy commentary. Three 2026 analyses of largely overlapping Federal IDR data each illustrate a different gate. These analyses of largely overlapping Federal IDR data illustrate the immediate relevance of this framework.

*Niskanen Center (July 2026): Causation* 

A widely read commentary [2] combines largely corroborated descriptive statistics (2.5 million 2025 disputes, a large provider win rate, median winning offers 4-5× QPA, concentrated filing among four organizations accounting for nearly half of all disputes) with a categorical causal claim that, without reform, patients will continue to bear IDR’s costs through higher premiums and slower wage growth rather than through the surprise bills the NSA was designed to eliminate [2]. CBO’s contemporaneous assessment of the same underlying data treats the premium question as unresolved and requests the market-level causal research needed to answer it [5].

Elevance Health (June 2026): Comparator

A payer-sponsored analysis of 7,304 planned-procedure claim lines [23] recommends preserving “the QPA’s role as the central statutory reference point,” while its own data show a mean QPA for these procedures of $798 against its own mean in-network paid-claims benchmark of $1,614 for the same codes. Because [23] is an Elevance organizational report and is not treated as independent confirmation of the Elevance-affiliated peer-reviewed analysis in reference [16], which appears to draw on the same underlying payer data. An insurer’s own QPA sitting at roughly half its own paid-claims figure illustrates the narrower principle that benchmark availability does not itself establish benchmark validity. This comparison does not establish that the QPA is generally inaccurate or that the resulting IDR awards are economically correct.

Health Affairs Forefront (March 2026): Selection

The most careful of the three sources [4] correctly distinguishes eligibility challenges from ineligibility determinations (40% challenged vs. 17% found ineligible) and conditions its own QPA-multiple finding explicitly on QPA’s accuracy. Its remaining vulnerability is selection: citing Virginia’s 45% state-system win rate against the approximately 85% federal rate as evidence that added guardrails improve outcomes treats two differently selected populations (different eligibility rules, claims mix, and benchmark construction) as if a single rule difference explains the gap [4].

Three actors with different incentives, working from overlapping public data, each illustrate a different gate. Each is otherwise credible, and each is discussed here only at the point where its own evidence does not support its conclusion.

Source Independence

Repeated use of the same upstream data does not constitute independent empirical replication. Also, [16] is an Elevance-affiliated peer-reviewed analysis of Elevance data, and [23] is an Elevance organizational analysis drawing on the same or substantially overlapping organizational data. We therefore do not treat [16] and [23] as independent confirmation of one another. More broadly, Elevance’s analysis [23] cites the Health Affairs Forefront analysis discussed above [4], while the Niskanen commentary [2] draws on CMS data [9], Brookings [1], and contemporary press rather than citing Elevance or Health Affairs Forefront directly. Several prominent analyses therefore rely materially on overlapping upstream Federal IDR data. This is a source-independence limitation, not evidence of coordination among the organizations involved.

Symmetric Evidentiary Burden, Restated

Every result above admits a mirror question. A provider win rate invites scrutiny of provider conduct, but an equivalent payer loss rate does not generate comparable scrutiny of payer-offer calibration or QPA construction. Awards above QPA invite scrutiny of arbitrators and providers, but QPA’s own accuracy is treated as a default rather than validated. Concentrated provider filing invites a gaming inference that an equivalent concentration statistic, applied to payer behavior, would not automatically receive absent the same exposure denominator this paper says is missing for providers. This paper does not answer any of these mirror questions in the anti-payer direction, as that would simply relocate the original error. The claim is narrower: the same Denominator, Comparator, Selection, Causation, and Attribution requirements should apply regardless of which party’s conduct is under discussion, and at present they frequently do not. Twelve incomplete inferences do not become one complete inference merely because they point in the same direction. Failure to satisfy a gate does not establish the opposite proposition; it limits the strength of inference that can validly be drawn from the evidence presently available.

Alternative Explanations for the Observed Adjudicatory Asymmetry

A hidden assumption in the reform narrative is that an insurer-reported QPA can be treated as presumptively accurate because calculation error is merely hypothetical. The Aetna audit demonstrates this premise cannot simply be assumed, in at least one documented instance [15,17]. Error exists, but prevalence and directionality are not established. This does not establish that QPA is generally wrong. Rather, it makes it difficult to sustain the opposite default: that QPA should be presumed correct until providers explain departures from it, rather than being independently confirmed by a regulator. Nor does this analysis assume certified IDR entities identify a uniquely correct market price. They apply a statutory decision rule considering QPA alongside permissible additional factors, and their selections are outcomes generated under that rule, not independent appraisals of fair market value. A benchmark’s statutory status does not establish its economic validity. QPA’s role as the factor adjudicators must always consider is a legal fact, not a demonstration that it reflects a correct commercial value. Persistent divergence between QPA and selected offers is therefore an empirical question to resolve, not evidence that either the QPA or the award is correct. Given that provider offers prevail in most fully contested determinations, that selected offers also exceed QPA in most determinations, and that these are substantially the same determinations, what combination of case selection, payer-offer strategy, benchmark performance, statutory factors, and adjudicator behavior produces that result? Payer-offer proximity to QPA itself did not materially resolve the asymmetry under the primary and wider-band specifications, and showed only a small positive association under the exact-match sensitivity specification.

Implications for QPA-Centered Reform

Federal IDR generates far more disputes than forecast, at rates regulators did not anticipate. Providers prevail in a strong majority of contested determinations, and provider victories substantially overlap with determinations in which selected payments exceed QPA. The magnitude of that overlap is consistent with the positioning of provider offers relative to QPA and should not, by itself, be treated as evidence of an independent adjudicatory mechanism. None of these observations currently establishes that the system is being exploited, that QPA is generally miscalculated, that arbitrators are malfunctioning, or that current award levels are materially inflating premiums. They equally do not establish the opposite: that current outcomes reflect accurate valuation, that no benchmark or process problems exist, or that no reform is warranted. Proposals to expand QPA’s determinative role in payment policy should therefore be evaluated using evidence capable of addressing denominator specification, comparator validity, selection, causation, and attribution. Such evidence would include a valid denominator for volume and concentration statistics, expanded regulatory or independent validation of QPA methodology, reason-specific eligibility data, symmetric measures of payer and provider behavior, and causal designs examining relationships between IDR intensity and subsequent prices, network participation, and premiums.

The evidentiary principles organized by the Five-Gate framework are not unique to Federal IDR. Denominator specification, comparator validity, selection, causal inference, and attribution are established considerations in observational evidence appraisal. The present study demonstrates one structured application of those principles to Federal IDR. Whether this organization provides a reliable, reproducible, or useful framework in other payment-policy settings requires independent application and evaluation.

Research Agenda

Table 5 sets out hypotheses in this debate, the data and design each requires, and the findings that would strengthen or weaken each. This is a prospective research agenda, not a pre-registered protocol.

**Table 5:** Empirical test and research matrix Prospective research agenda identifying testable hypotheses arising from the Federal IDR debate, the data and study design required to evaluate each hypothesis, and findings that would strengthen or weaken it. These proposed analyses are research priorities, not completed studies or a pre-registered protocol. Current-status entries draw on the present analysis (QPA proximity row), CMS data [9,21] (adjudicator-specific effects and QPA calculation error rows), and CBO [5] (network exit, negotiated-price inflation, and premium inflation rows); the provider self-selection row is untested and has no source data available. CMS: Centers for Medicare & Medicaid Services; QPA: Qualifying Payment Amount; IDR: Independent Dispute Resolution; CBO: Congressional Budget Office

Hypothesis	Data needed	Design	Finding that would strengthen the hypothesis	Finding that would weaken the hypothesis	Current status
QPA proximity independently predicts provider victory	Line-item offer/QPA + outcome	Stratified MH / regression, cluster bootstrap CI	OR meaningfully >1 after adjustment	OR ~1 after adjustment	Little evidence of a material association in the primary and wider-band specifications; small association in the exact-match sensitivity analysis (OR 1.037, 95% CI (1.002, 1.080))
Provider self-selection alone explains win rate	Case-level filing decisions, declined disputes	Selection model	Win rate fully explained by observable case strength	Residual asymmetry after controlling for case strength	Untested (data not available)
Adjudicator-specific effects	Certified IDR entity identifier + outcomes	Case-mix-adjusted win rate by entity	Persistent entity-level residual after adjustment	No persistent residual	Untestable before Q3/Q4 2025 public-use file (entity field now available)
Network exit due to IDR	Market-level network participation over time	Difference-in-differences	Participation falls in high-IDR-intensity markets	Participation stable or rises	Preliminary evidence against (CBO)
Negotiated-price inflation from IDR	Market-level IDR intensity + subsequent prices	Event study / DiD	Prices rise faster in high-IDR markets	No differential price growth	Untested; CBO explicitly requests this study
Premium inflation from IDR	Premium data + IDR intensity	Multi-year panel	Premiums rise with IDR intensity	No relationship	CBO: evidence mixed, unresolved
QPA calculation error is common	Full audit sample across payers	Expanded CMS audit program	Errors found in most audited payers	Errors rare or isolated	Untested (1 of 25 initiated audits completed as of last reporting)

The research agenda includes six priorities. First, an independent QPA audit should examine the contract populations, specialty classification, and geographic methodology underlying sampled QPAs, conducted by regulators or an independent auditor. Second, CMS should construct and publish an eligible-claims denominator that would permit exposure-normalized measurement of filing intensity by organization and payer. Third, reason-specific ineligibility reporting (distinguishing cooling-off, jurisdiction, plan type, information deficiency, and other categories) should replace the single collapsed "ineligible" category. Fourth, a payer initial-payment analysis should examine insurer initial payment as a fraction of QPA, prior contracted rate, and eventual award, to test whether payer-specific initial-payment patterns predict arbitration escalation. Fifth, a risk-adjusted, IDR-entity-level win-rate analysis should be conducted using the newly available certified IDR entity identifier field [21]. Sixth, a market-level difference-in-differences analysis should compare IDR intensity against subsequent network participation and negotiated prices.

**Figure 5:**
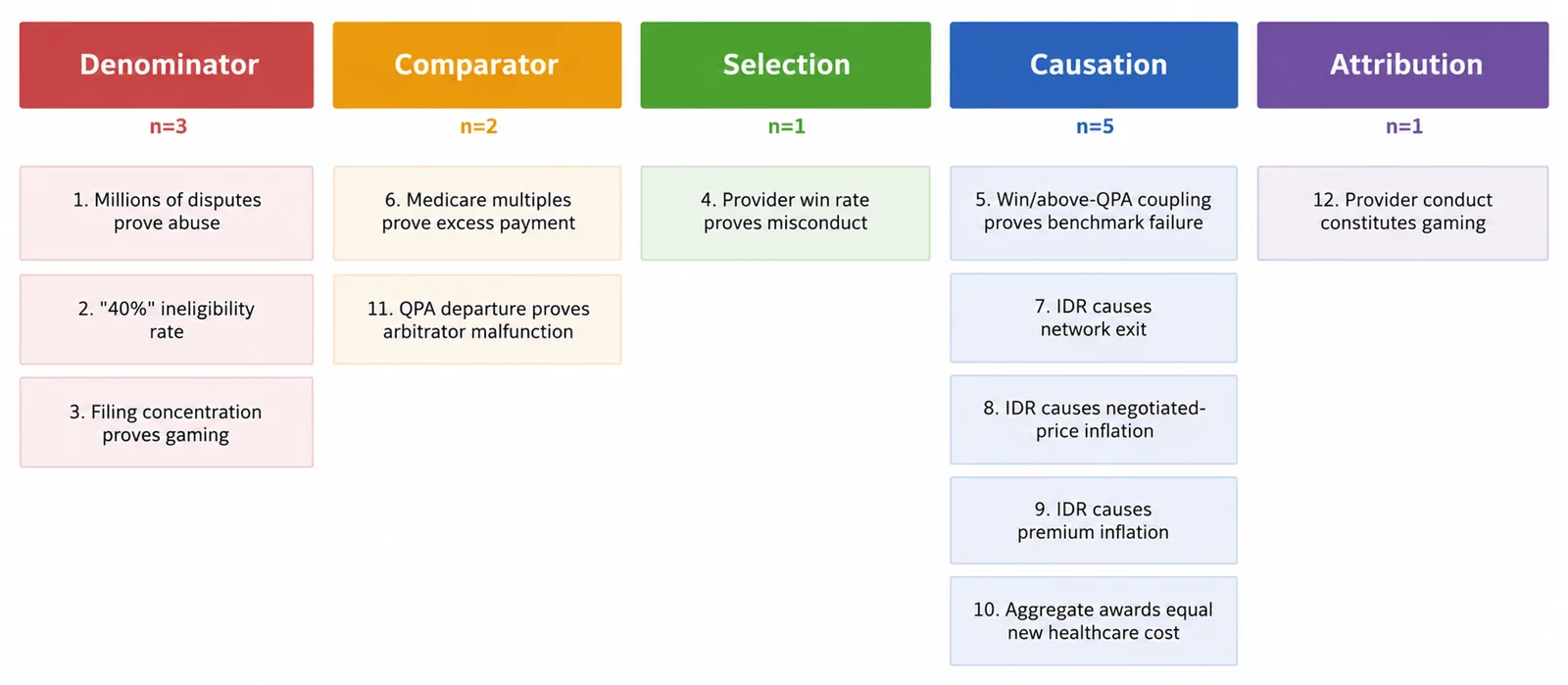
Distribution of Federal IDR claims by primary unmet evidentiary gate The 12 recurring Federal IDR claims evaluated in this study are grouped by the primary unmet evidentiary gate identified for each claim. Denominator includes three claims, Comparator two, Selection one, Causation five, and Attribution one. Several claims implicate more than one gate; the figure displays only the primary unmet gate for visual clarity. Table 1 provides the full multi-gate analysis and source citations. IDR: Independent Dispute Resolution

The preceding sections examine the 12 claims gate by gate. Table 6 summarizes what each headline statistic establishes, the stronger claim it is commonly used to support, and the evidentiary gate that remains uncleared. Figure 5 provides a complementary visual summary of the 12 claims grouped by their primary unmet evidentiary gate.

**Table 6:** What the evidence supports The preceding sections examine the 12 claims gate by gate. This table compresses that analysis into a single reference: for each headline statistic in the Federal IDR debate, what it establishes on its own terms, what it is commonly used to claim, and the specific gate that claim has not yet cleared. This is a synthesis of the present analysis (Table 1 and Results), drawing on CMS data [9], CBO [5], and the Aetna QPA audit findings [15,17]. CMS: Centers for Medicare & Medicaid Services; QPA: Qualifying Payment Amount; IDR: Independent Dispute Resolution

Headline statistic	What it establishes	What it is often used to claim	Gate not yet cleared
6.3M+ disputes initiated through May 2026	Utilization vastly exceeds the original ~22,000/year forecast	The system is being abused	Denominator — no public count of eligible-but-unfiled claims exists to compute a rate
16.83% of initiated disputes found ineligible	A real, non-trivial ineligibility rate, well below the ~40% challenge rate some cite as if it were the same figure	Provider misconduct at scale	Denominator — ineligibility is procedural in substantial part (CMS’s own 2026 rule targets information-exchange failures, not provider conduct)
A handful of organizations file a large share of disputes	Filing is concentrated	Those organizations are gaming the system	Denominator — no eligible-claims-per-organization exposure denominator exists to establish disproportion
87% non-default H1 provider win rate	A large, real adjudicatory asymmetry	Arbitrators or providers are behaving improperly	Selection — the asymmetry’s cause (case selection, offer strategy, benchmark performance, adjudicator effects) is undetermined, and the population of eligible-but-unfiled claims is unobserved
97.67% of provider offers exceed QPA; 97.95% of provider wins produce above-QPA awards	These are substantially the same phenomenon, not two independent findings	Arbitrators are systematically inflating awards above QPA	Causation — the observed coupling is substantially consistent with offer positioning; no independent adjudicatory mechanism has been isolated
Payer-offer proximity to QPA vs. provider victory: primary OR 1.0064 (0.973, 1.045); exact-match OR 1.0367 (1.002, 1.080)	Little evidence of a material association in two of three specifications; a small association in the third	QPA “explains” or “fails to explain” the win rate in an absolute sense	Causation — an association analysis under one stratification scheme, not a causal design; unmeasured covariates remain possible
Awards reported as multiples of Medicare	A mathematical distance from a statutorily excluded benchmark	Economic excess in the award	Comparator — Congress barred Medicare from adjudication; a large multiple of an inapplicable benchmark does not establish valuation error
CMS’s one completed QPA audit found 5 miscalculated QPAs (Aetna, both directions) [15,17]	QPA calculation error can occur in operational practice	QPA is generally unreliable, or generally accurate	Comparator — existence of error demonstrated; prevalence and directionality not established from one audit
CBO: preliminary evidence of rising network participation, generally stable/declining prices	Some evidence against the strongest form of the network-exit and price-inflation hypotheses	Federal IDR has (or has not) caused network or price effects	Causation — CBO itself characterizes this evidence as preliminary and has requested the market-level research that would settle it
Billions in gross annual award/administrative totals	Real payment and administrative resource flows exist at scale	Federal IDR is adding billions in new healthcare costs	Causation — gross flows are not the same quantity as the incremental payment attributable to arbitration relative to a non-arbitration counterfactual
99.9% of disputes initiated by providers (Q4 2025)	Providers overwhelmingly control initiation	Provider conduct constitutes gaming of the process	Attribution — initiation is provider-controlled, but the certified IDR entity, not the provider, selects the prevailing offer; the aggregate initiation share does not establish who controlled the outcome

Limitations

This is a structured evidentiary evaluation with an original secondary-data analysis; it is not a causal econometric analysis. No public national denominator of federally IDR-eligible out-of-network claims exists, so this study cannot estimate an abuse rate in either direction. Adjudicated disputes are a highly selected population, and these findings should not be generalized to all commercial out-of-network claims. This study does not independently validate QPA calculations, payer or provider offers, or IDR determinations. Evidence concerning network participation, negotiated prices, and premiums remains temporally limited, and CBO itself characterizes much of it as preliminary. Advocacy statements were included to identify claims under active debate, not as a systematic corpus representative of all payer or provider positions. Exact CMS-reported payer strings were used without payer-name canonicalization, so economically related payer-name variants may remain divided among separate strata; no payer-name-normalized analysis was performed. The deterministic extraction and stratification pipeline was subsequently reconstructed directly from the original CMS Q1-Q2 2025 source workbooks using preserved code, and the principal analytic artifacts reproduced exactly by SHA-256 comparison. The original bootstrap implementation and random seed were not recoverable, so the reported confidence intervals were independently corroborated rather than reproduced bit-for-bit.

The Five-Gate Evidentiary Framework has limitations independent of the underlying Federal IDR data. It has not undergone external validation, and this study does not establish that its five categories are exhaustive, mutually independent, superior to alternative evidence-appraisal approaches, or reproducibly classified by independent users. Application of individual gates may require judgment regarding what constitutes an adequate denominator, comparator, causal design, or attribution analysis. The framework should therefore be understood as a proposed structured analytic heuristic rather than a validated methodological instrument. Future work should evaluate operational definitions, inter-rater reliability, and independent application in other policy settings. Finally, the conclusions of this study concern the evidentiary sufficiency of the statistics examined. Failure of the available evidence to establish a proposed mechanism or policy effect should not be interpreted as evidence that the mechanism or effect does not exist.

## Conclusions

This paper asked whether the Federal IDR statistics most commonly cited in the reform debate support the policy inferences drawn from them. The examined statistics establish substantial utilization, ineligibility, filing concentration, adjudicatory asymmetry, and divergence from QPA, but do not independently establish provider abuse, benchmark validity, network erosion, price or premium inflation, or the incremental cost of arbitration. These conclusions concern the limits of the evidence examined; they should not be interpreted as evidence that the corresponding mechanisms or effects do not exist.

The supplemental H1 2025 line-item analysis found that provider victory and above-QPA payment substantially overlap and that provider offers themselves exceed QPA in nearly all analyzed line items. Within the analyzed Federal IDR disputes, the observed association between payer-offer proximity to QPA and provider victory was small and sensitive to specification. The primary and wider-band specifications showed little evidence of a material association, while the post-hoc exact-match sensitivity specification showed a small positive association that is exploratory rather than confirmatory. These findings do not identify the mechanism responsible for the provider-payer win-rate asymmetry, which remains unresolved.

Applied symmetrically, the same evidentiary requirements that prevent provider win rates from proving provider virtue also prevent those rates from proving provider abuse. Policy decisions increasing QPA’s determinative role require evidence capable of distinguishing case selection, payer and provider offer behavior, benchmark performance, statutory-factor evidence, and adjudicatory effects. The Five-Gate Evidentiary Framework offers one structured way to organize those questions using established principles of evidence appraisal. It is a proposed framework rather than a validated methodological instrument, and its reliability, completeness, and generalizability in other payment-policy settings require independent evaluation.
